# Effect of Immersive Virtual Reality on Chemotherapy-Related Side Effects in Patients Receiving Paclitaxel-Carboplatin With or Without Bevacizumab: 2-Arm Randomized Controlled Trial

**DOI:** 10.2196/65924

**Published:** 2025-08-14

**Authors:** Kazuyuki Niki, Satoshi Nakagawa, Misaki Arai, Ayaka Morimoto, Yutaka Ueda

**Affiliations:** 1 Department of Clinical Pharmacy Research and Education, The University of Osaka Graduate School of Pharmaceutical Sciences, Yamadaoka 1-6, Osaka, Suita, 565-0871, Japan, 81 6-6879-8250; 2 Department of Obstetrics and Gynecology, The University of Osaka Graduate School of Medicine, Suita, Osaka, Japan

**Keywords:** virtual reality, paclitaxel/carboplatin therapy, supportive care, randomized controlled trial, immersive virtual reality, iVR, gynecologic cancer, paclitaxel, carboplatin, pain, anxiety, RCT, nausea, depression, antiemetic medications

## Abstract

**Background:**

Symptomatic drug treatment is generally used to treat various side effects associated with paclitaxel-carboplatin (TC) or TC plus bevacizumab (TC+Bev). However, this can lead to increased adverse effects from additional drugs. Immersive virtual reality (iVR) reduces pain and anxiety.

**Objective:**

This study aimed to investigate the efficacy of iVR in managing side effects associated with TC or TC+Bev therapy.

**Methods:**

This 2-arm randomized controlled trial included patients with gynecologic cancer scheduled to undergo their first course of TC/TC+Bev. Patients in the intervention group received iVR for approximately 10 minutes/day for 7 consecutive days, starting on the first day of treatment. The primary endpoint was the severity of physical and psychiatric symptoms measured using the Japanese version of the revised Edmonton Symptom Rating System (ESAS-r-J). The secondary endpoint included the proportion of patients who used additional antiemetic medications, the complete response (CR) rate to nausea, and the severity of anxiety, measured using the state-trait anxiety inventory-JYZ (STAI) Y-1. Patients in the nonintervention group received supportive and symptomatic treatments.

**Results:**

The analysis included 28 and 30 patients in the intervention and nonintervention groups, respectively. The change in ESAS-r-J scores between days 1 and 7 and nausea were significantly worse in the intervention group on day 4 only (*P*<.001); however, the nonintervention group showed significantly worse scores on days 3, 4, and 5. Depression was not significantly worse in the intervention group on any day other than on day 1; however, the nonintervention group showed significantly worse scores on day 4. The proportion of patients who used additional antiemetic medications from days 2 to 7 was significantly lower in the intervention group than in the nonintervention group (*P*=.02). Regarding the change in STAI Y-1 on day 1 of TC or TC+Bev therapy, the mean score was significantly lower after the iVR experience than before the experience in the intervention group (from 43.8 to 34.8; *P*<.001), whereas, in the nonintervention group, no significant difference was observed before and after anticancer drug administration (from 44.9 to 43.9; *P*=.54).

**Conclusions:**

iVR may reduce the deterioration of nausea and depression more effectively in patients with gynecologic cancer undergoing TC or TC+Bev therapy than in those undergoing nonintervention, especially in delaying the onset of nausea and accelerating recovery.

## Introduction

Paclitaxel-carboplatin therapy (TC therapy) or TC plus bevacizumab (TC+Bev) therapy is the first-line chemotherapy for ovarian cancer [1]. However, it often causes side effects, including digestive symptoms, myalgia, arthralgia, and fatigue [2], which can lead to anxiety and even abandonment of treatment. Therefore, implementing appropriate preventive measures is important. In addition, the increased risk of side effects owing to additional drug intake and associated medical costs has been viewed as a problem. Therefore, a safe and economical nondrug-based approach is desirable.

In recent years, digital therapeutics (DTx) has attracted attention as a new nondrug approach. Although DTx requires maintenance for security and other safety and quality issues, its advantages include practical use with a smartphone, lower development costs than drugs, and a lower risk of side effects. We focused on virtual reality (VR) among the digital devices used in DTx. VR is a technology that works on the human sensory organs to artificially create an environment that feels like reality. Since the commercial availability of a simple immersive VR (iVR) device in 2016, medical applications of iVR have rapidly advanced, demonstrating effectiveness in reducing intractable chronic pain that has not responded to drugs [3] and reducing anxiety in hospitalized patients with breast cancer [4]. We observed that various physical and mental symptoms, including pain, anxiety, and depression, in patients with terminal cancer were temporarily improved by traveling to their desired locations, such as their homes or memorable places, via iVR [5]. Although these symptoms are common side effects of TC therapy, previous evaluations focused on the transient effects of a single intervention and did not examine the sustained effects of iVR with continuous intervention. Therefore, we conducted a 7-day continuous iVR intervention in patients with gynecological cancer undergoing TC or TC+Bev therapy to evaluate its efficacy.

## Methods

### Participants

This study included patients undergoing TC or TC+Bev therapy who were hospitalized at the Department of Obstetrics and Gynecology, Osaka University Hospital. Patients were excluded if they were younger than 20 years, visually impaired, hearing-impaired, prone to motion or visual sickness, had difficulty sitting up, or had cognitive decline to the point of being unable to answer the questionnaire.

### Study Design

This single-center, open-label, 2-arm, randomized controlled trial was conducted using the substitution block method. This study was conducted in line with the CONSORT (Consolidated Standards of Reporting Trials) guidelines (the CONSORT checklist is provided in Checklist 1).

### Evaluation Items and Methods

Patient characteristics were collected from medical records and included age, sex, Eastern Cooperative Oncology Group Performance Status (ECOG PS), primary cancer site, metastatic cancer site, cancer recurrence, date of first cancer diagnosis, disease duration, surgery for cancer before chemotherapy, history of upper limb numbness before the study, and concomitant medications in the first week after starting anticancer therapy. Opioids, nonsteroidal anti-inflammatory drugs (NSAIDs), and acetaminophen were included in the study as analgesics according to the World Health Organization’s 3-step analgesic ladder. Patient anxiety was assessed using the Japanese version of the State-Trait Anxiety Inventory (STAI) [6,7], which consisted of 2 questionnaires: STAI Y-1 and Y-2. STAI “Y” means that this is a new, revised version of the original STAI “X.” The STAI Y-1 examines transient situational reactions (state anxiety) to an anxiety-provoking event, “How you are feeling right now,” using a 4-point scale from 1 (not at all true) to 4 (very true). The STAI Y-2 tests relatively stable reactions to anxious experiences (trait anxiety) by asking “How do you usually feel in general?”. Each questionnaire had 40 questions, categorized into the presence (P-scale) and absence (A-scale) of anxiety, with 20 questions in each category (10 questions per scale). The side effects associated with TC therapy were assessed using the Japanese version of the Edmonton Symptom Assessment System (ESAS-r-J) [8]. The ESAS-r-J is a questionnaire that assesses 9 symptoms frequently experienced by patients eligible for palliative care (pain, tiredness, drowsiness, nausea, lack of appetite, shortness of breath, depression, anxiety, and well-being) on an 11-point scale from 0 (no symptoms) to 10 (most severe symptoms). Dizziness and headache were assessed using a numerical rating scale (NRS), an 11-point scale ranging from 0 (not at all) to 10 (most severe). Furthermore, fun and happiness were assessed using NRS from 0 (not at all) to 10 (feeling quite a bit) [9]. To investigate their perceptions of the iVR experience, a questionnaire adapted from Johnson T et al [10] was used. The questionnaire included questions such as, “How easy was it to learn how to operate this VR program?” “How confident would you be in recommending this VR program to a friend experiencing a similar situation?” and “How useful do you think this program has been for you?” and “Were there any difficulties you experienced during this session?” Symptoms experienced in 7 days before the start of the second course were assessed using the Japanese version of the integrated palliative outcome scale (IPOS-J) [11]. The IPOS consists of 10 questions about the patient’s difficulties, physical symptoms (pain, shortness of breath, weakness or lack of energy, nausea, vomiting, poor appetite, constipation, mouth problems, drowsiness, and poor mobility), and feelings such as anxiety levels of the patient and family. Responses are rated on a 5-point scale from 0 (mildest symptoms/smallest problems) to 4 (most severe symptoms/greatest problems).

### iVR Operation

**Figure 1.**
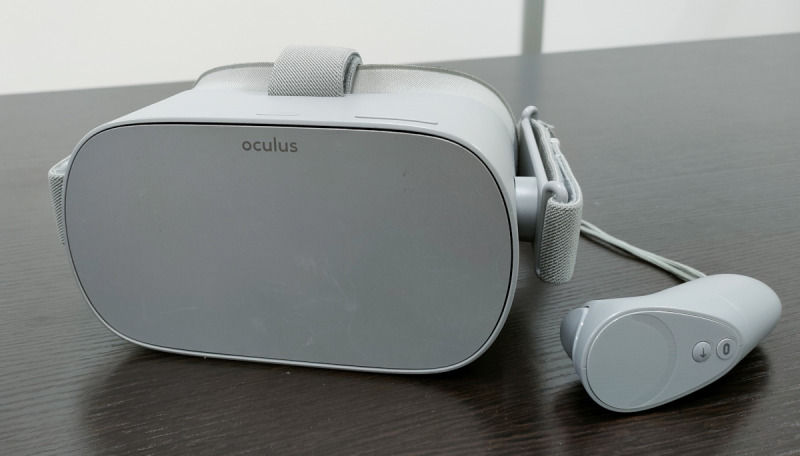
Virtual reality head-mounted display used in this study.

Oculus Go (Meta Platforms, Inc) was used as the VR head-mounted display (Figure 1). A pamphlet was prepared alongside the iVR experience (Figure S1 in Multimedia Appendix 1), and the VR content was selected according to the patient’s needs. The pamphlet included a portion of the video to make it easy to understand what could be experienced with each app, as well as descriptions of the necessity and operational complexities. The apps introduced in the brochure were Wander (Parkline Interactive, LLC), Ocean Rift (Picselica Ltd), YouTube VR (Google LLC), HOMESTAR VR (The Pocket Company), ART PLUNGE (Space Plunge), Disney VR (Disney), Jurassic World: Blue (Felix & Paul Studios), Cirque du Soleil VR (CIRQUE DU SOLEIL). The details of these applications are provided in Table S1 in Multimedia Appendix 2. The iVR images viewed by the patients were mirrored on the tablets for research purposes. The patients were instructed on how to operate the controllers. For those who had difficulty operating the controller, a researcher (KN) assisted them in viewing the mirrored images.

### Study Schedule

Evaluations were conducted for both groups during the 7 days following the administration of TC or TC+Bev and at the next visit. The details of the TC and TC+Bev regimens are shown in Table 1. Three antiemetic agents were administered to all the patients, granisetron (a 5-HT3 receptor antagonist), aprepitant (an NK-1 receptor antagonist), and dexamethasone. The doses of each drug are listed in Table 1. The evaluation schedule for each group was as follows.

**Table 1.** Paclitaxel-carboplatin or paclitaxel-carboplatin plus bevacizumab therapy regimen.

Medicine (administration route)	Day 1	Day 2	Day 3	Day 4	Day 5
Diphenhydramine Hydrochloride (orally)	50 mg				
Aprepitant (orally)	125 mg	80 mg	80 mg		
Dexamethasone	9.9 mg (iv)				
Granisetron (iv^a^)	3.0 mg				
Famotidine (iv)	20 mg				
Paclitaxel (iv)	175 mg/m^2^				
Carboplatin (iv)	AUC^b^=5				
Ramosetron hydrochloride (orally)		0.1 mg	0.1 mg	0.1 mg	0.1 mg
Bevacizumab (iv) ※Only TC+Bev therapy	15 mg/kg				

a iv: intravenous.

b AUC: area under the curve.

### Intervention Group

On day 1, the iVR intervention and evaluation were performed during carboplatin administration. Patients first responded to the STAI Y-1 and Y-2 questionnaires and then selected the iVR app they wanted to use from the brochures. The iVR intervention was conducted for approximately 10 minutes, and the ESAS-r-J, NRS, and STAI Y-1 scores were recorded. We set the duration of the iVR viewing time to 10 minutes to avoid burdening the patients because Chen et al [12] reported that the longer the viewing time, the higher the risk of VR sickness, and we have heard patients say that “I felt the VR headset was getting heavier and heavier” after 10 minutes or more in our previous studies [5]. On days 2‐6, the iVR intervention was conducted in the same manner as on day 1, and the ESAS-r-J and NRS scores were recorded. On day 7, the iVR intervention was conducted; the ESAS-r-J, NRS, and STAI Y-2 scores were recorded; and the patients completed the VR experience questionnaires. At the second visit, the ESAS-r-J, NRS, and IPOS-J scores were recorded (for 7 days).

### Nonintervention Group

On day 1, the drugs were administered according to the usual TC or TC+Bev regimen, and the patients completed the STAI Y-1 and Y-2 questionnaires 30 minutes before receiving carboplatin. ESAS-r-J, NRS, and STAI Y-1 scores were recorded during carboplatin administration. On days 2‐6, ESAS-r-J and NRS scores were recorded at a fixed time each day. On day 7, the ESAS-r-J, NRS, and STAI Y-2 scores were recorded at the same time. At the second visit, the ESAS-r-J, NRS, and IPOS-J scores were recorded (for 7 days).

### Primary and Secondary Endpoints

The primary endpoint was the change in ESAS-r-J scores from days 1 to 7. The secondary endpoints were the change in the NRS scores for positive feelings (fun, happiness, and anticipation for the next VR experience) and adverse reactions (dizziness and headache), the change in the STAI Y-1 scores before and after the first iVR experience and in STAI Y-2 scores between days 1 and 7, questionnaire description of the VR experience on day 7, complete response (CR) rate for acute, delayed, and anticipatory vomiting, IPOS-J scores before the second course of administration, and the outpatient transfer rate for the second course of administration. CR was defined as the absence of additional medication (antiemetics) or emesis. Acute nausea was defined as nausea occurring 0‐24 hours after carboplatin administration, delayed nausea was defined as nausea occurring 24 hours-5 days after carboplatin administration, and anticipatory nausea was defined as the use of additional antiemetic drugs immediately before the next administration [13,14].

### Statistical Analysis

The Bell Curve for Excel (Social Survey Research Information, Japan) was used for the analysis. Statistical significance was set at *P*<.05. Student *t* test, Fisher exact probability test, chi-square test, and Mann-Whitney *U* test were used to compare patient characteristics. The Dunnett test was performed to compare the changes in the ESAS-r-J and NRS scores from days 2 to 7 relative to the baseline scores. Paired *t* tests were performed for comparisons of STAI Y-1 scores before and after day 1 of the iVR experience, and for STAI Y-2 scores on days 1 and 7. Student *t* tests were performed to compare STAI scores between the 2 groups. The Fisher exact test was performed to determine the CR rates for acute, delayed, and anticipatory emesis. Student *t* tests were performed to compare the mean IPOS-J scores before the administration of the second course. The chi-square test was used to determine the outpatient transition rate for the second course.

### Sample Size

In our previous study [5], the effect size of the ESAS-r-J scores for anxiety and depression before and after the iVR experience was ≥0.8. Although the single-arm design of this study differed from those of previous studies, we aimed to achieve the same effect size for the ESAS-r-J scores for anxiety and depression. Therefore, the sample size was calculated to detect an effect size of 0.8 with a significance level of 5% and a power of 80%, resulting in a minimum number of 26 patients per group. Assuming that approximately 10% (3‐4 patients) dropped out during the study, the sample size was set at 30 patients per group, for a total of 60 patients.

### Ethical Considerations

The study was explained in writing by the physicians (YU or SN), and informed consent was obtained in written form from the patients. This study was conducted in compliance with the Declaration of Helsinki and Ethical Guidelines for Medical Research Involving Human Subjects, and was approved by the Ethical Review Committees for Interventional Research of Osaka University Hospital (approval number: 20002) and Clinical Research of Osaka University Graduate School of Pharmaceutical Sciences and Faculty of Pharmaceutical Sciences (approval number: Yakuhito 2020‐9). This study was registered in the UMIN Clinical Trials Registry (UMIN000041067).

## Results

### Participant Grouping and Background

**Figure 2.**
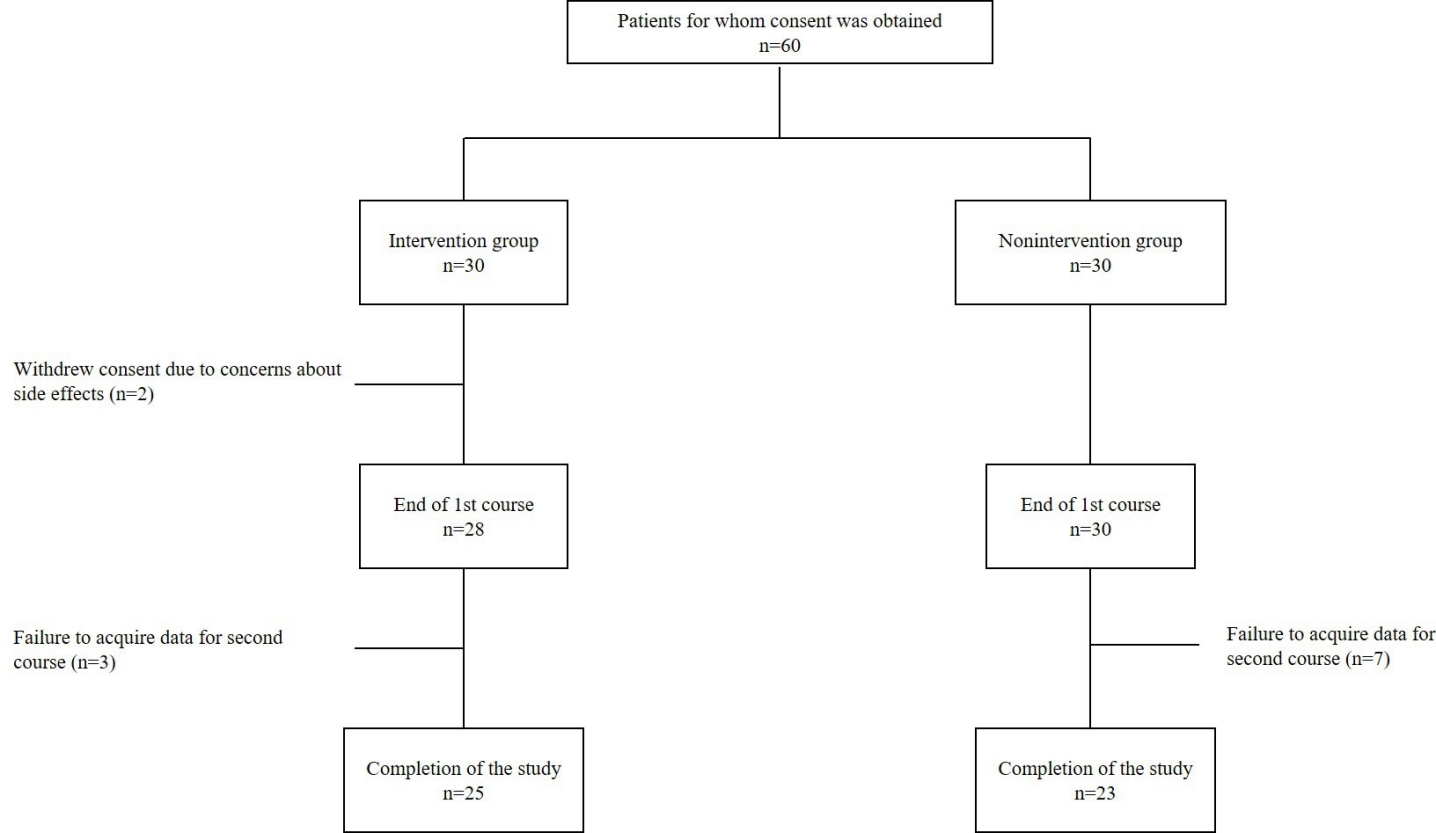
Selection process of the study patients.

Consent was obtained from 60 patients. Of these, 30 were assigned to the intervention group and 30 to the nonintervention group. A total of 2 patients withdrew their consent because they were concerned about continuing the iVR experience given the side effects of anticancer drugs. Consequently, 28 patients in the intervention group and 30 patients in the nonintervention group completed the 7-day study. Data could not be obtained at the second visit for three and 7 patients in the intervention and nonintervention groups, respectively, for the following reasons: the timing of the questionnaire could not be adjusted due to the transition to outpatient day chemotherapy, completing the questionnaire according to the schedule was deemed impossible due to the unavailability of the researcher, or patients could not be discharged from the hospital due to prolonged side effects (Figure 2). For the items evaluated during the 7-day study, the results of 28 and 30 patients in the intervention and nonintervention groups, respectively, who completed the study during this period without problems, were included in the analysis. For the items evaluated during the second hospital visit, the results of 25 and 23 patients who completed the study in the intervention and nonintervention groups, respectively, were included in the analysis. Furthermore, patients who were unable to respond to the questionnaire due to side effects or other reasons were included in the data analysis for the days when responses were obtained. Details of the patient characteristics are presented in Table 2. No significant differences were observed between the 2 groups.

**Table 2.** Patients’ characteristics.

Variables		Intervention group (n=28)	Nonintervention group (n=30)	*P* value
Age (years), mean (SD)		57.0 (10.3)	57.6 (11.3)	.85^a^
ECOG PS^b^, n (%)				>.99^c^
	0	25 (89.3)	27 (90)	
	1	3 (10.7)	3 (10)	
Primary cancer site, n (%)				.16^d^
	Ovary	20 (71.4)	22 (73.3)	
	Body of the uterus	5 (17.9)	1 (3.3)	
	Peritoneum	3 (10.7)	2 (6.7)	
	Neck of the uterus	0 (0)	3 (10.0)	
	Fallopian tube	0 (0)	1 (3.3)	
	Unknown	0 (0)	1 (3.3)	
Site of cancer metastasis, n (%)				.75^d^
	Peritoneal dissemination	8 (28.6)	6 (20)	
	Lymph node	7 (25.0)	7 (23.3)	
	Female genitalia	3 (10.7)	1 (3.3)	
	Lung	1 (3.6)	2 (6.6)	
	Large bowel	1 (3.6)	1 (3.3)	
	Nervous system	1 (3.6)	0 (0)	
	Diaphragm	1 (3.6)	0 (0)	
	Bone	0 (0)	1 (3.3)	
Disease duration (days), median (IQR)		62.0 (47.8‐77.8)	49.0 (40.3‐64.5)	.14^e^
Cancer recurrence, n (%)				.73^e^
	+	5 (17.9)	4 (13.3)	
	–	23 (82.1)	26 (86.7)	
Surgery before chemotherapy, n (%)				>.99^c^
	+	24 (81.8)	26 (86.7)	
	–	4 (18.2)	4 (13.3)	
Upper limb numbness, n (%)				.19^c^
	–	12 (42.9)	19 (63.3)	
	+	16 (57.1)	11 (36.7)	

a Student *t* test.

b ECOG PS: Eastern Cooperative Oncology Group Performance Status.

c Fisher exact probability test.

d Chi-square test.

e Mann-Whitney *U* test.

### Changes in ESAS-R-J Score

**Figure 3.**
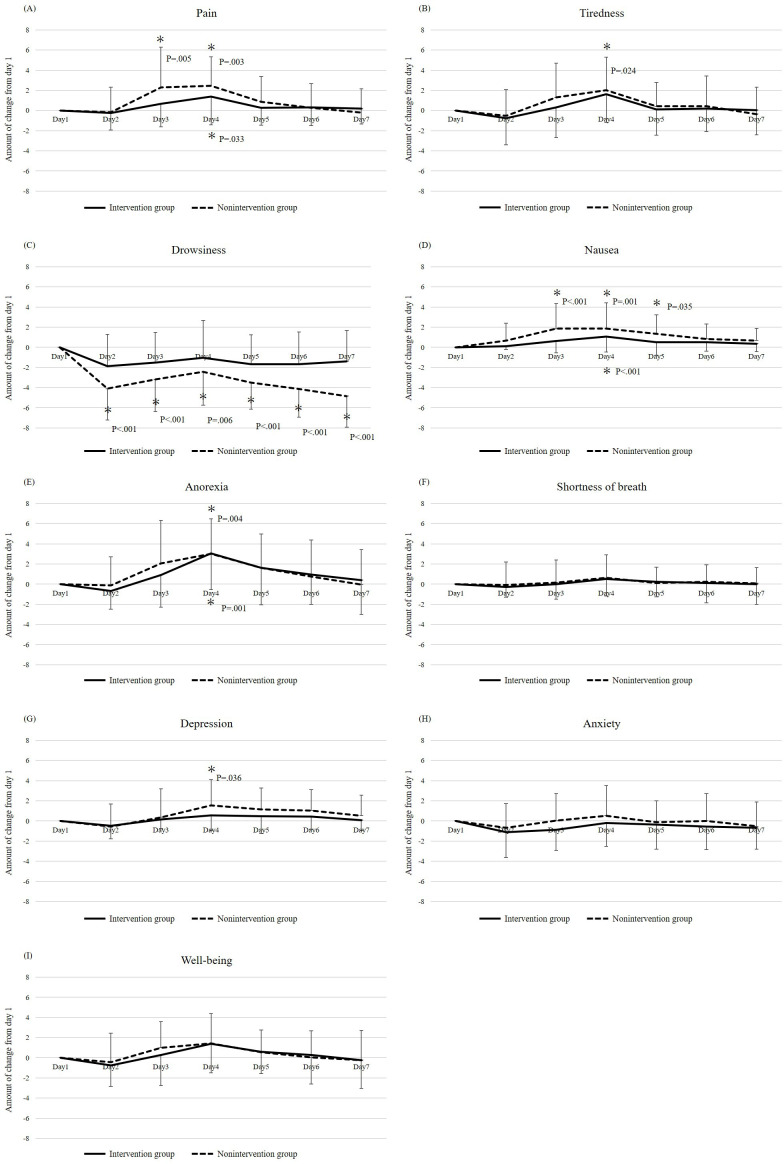
Changes in the Japanese version of the revised Edmonton Symptom Assessment System score from day 1 (day X–day 1) Pain (A), Tiredness (B), Drowsiness (C), Nausea (D), Anorexia (E), Shortness of breath (F), Depression (G), Anxiety (H), Well-being (I). *P<.05.

The mean change in the ESAS-r-J scores (days X–day 1) from days 2 to 7 relative to day 1 is shown in Figure 3. For pain, significantly greater changes in the score (worse symptoms) were observed on day 4 (*P*=.03) in the intervention group, and on days 3 (*P*=.005) and 4 (*P*=.003) in the nonintervention group (Figure 3A). For tiredness, significantly greater changes in the score were observed on day 4 (*P*=.02) in the nonintervention group, compared with no change in the intervention group (Figure 3B). Regarding drowsiness, significantly smaller changes (symptom improvement) were observed on days 2 (*P*<.001), 3 (*P*<.001), 4 (*P*=.006), 5 (*P*<.001), 6 (*P*<.001), and 7 (*P*<.001) in the nonintervention group. Conversely, no day in the intervention group was significantly worse (Figure 3C). For nausea, significantly greater changes were observed on day 4 (*P*<.001) in the intervention group and on days 3 (*P*<.001), 4 (*P*=.001), and 5 (*P*=.04) in the nonintervention group (Figure 3D). For anorexia, significantly greater changes were observed on day 4 in the intervention group (*P*=.001) and on day 4 in the nonintervention group (*P*=.004, Figure 3E). For depression, a significantly greater change was observed on day 4 in the nonintervention group, but no significantly greater change was observed on any day in the intervention group (Figure 3G). For shortness of breath, anxiety, and well-being, significantly greater changes were not observed on any day in either group (Figures 3F, 3H, and 3I).

### Complete Response Rates for Acute, Delayed, and Anticipatory Emesis

The CR rates in the acute phase were 96.4% in the intervention group and 93.3% in the nonintervention groups, respectively (*P*>.99). The delayed CR rates were 64.3% and 40% in the intervention and nonintervention groups, respectively (*P*=.074). The predictive CR rates were 100% and 95.7% in the intervention and nonintervention groups, respectively (*P*>.99).

### Percentage of Patients Who Used an Additional Dose of Medication

Throughout the 7-day period, the proportion of participants who received additional doses of antiemetic was smaller in the intervention group than in the nonintervention group (Table 3). In particular, after the acute phase (days 2‐7), the average percentage of additional doses was significantly lower in the intervention group than in the nonintervention group (*P*=.02). The additional antiemetic drugs are listed in Table S2 in Multimedia Appendix 3. The analgesic uses are summarized in Table 3. None of the participants used opioids. Only loxoprofen was used as NSAIDs. There was no significant difference between the 2 groups in the number of participants who used acetaminophen or loxoprofen during the 7-day period. Antidepressants were administered to 1 participant in the intervention group (aripiprazole) and 1 participant in the nonintervention group (quetiapine), both of which were administered regularly. No participants in either group used benzodiazepines.

**Table 3.** Additional medication during the first course of antiemetic and analgesics use.

		Intervention group	Nonintervention group	*P* value
Antiemetic use, n (%)				.02
	Day 1	1 (3.6)	2 (6.7)	
	Day 2	2 (7.1)	5 (16.7)	
	Day 3	6 (21.4)	13 (43.3)	
	Day 4	6 (21.4)	15 (50.0)	
	Day 5	6 (21.4)	16 (53.3)	
	Day 6	7 (26.9)	12 (40.0)	
	Day 7	4 (18.2)	8 (27.6)	
	Delayed phase (days 2-7), median (IQR)	6.0 (4.5-6.0)	12.5 (9.0-14.5)	
Analgesics use, n (%)				.83
	Day 1	5 (17.9)	4 (13.3)	
	Day 2	24 (14.3)	5 (16.7)	
	Day 3	11 (39.3)	14 (46.7)	
	Day 4	12 (42.9)	20 (66.7)	
	Day 5	11 (39.3)	19 (63.3)	
	Day 6	6 (21.4)	15 (50.0)	
	Day 7	4 (14.3)	9 (30.0)	

### Change in NRS Score for Positive Feeling and Adverse Reactions Associated With the iVR Experiences

**Figure 4.**
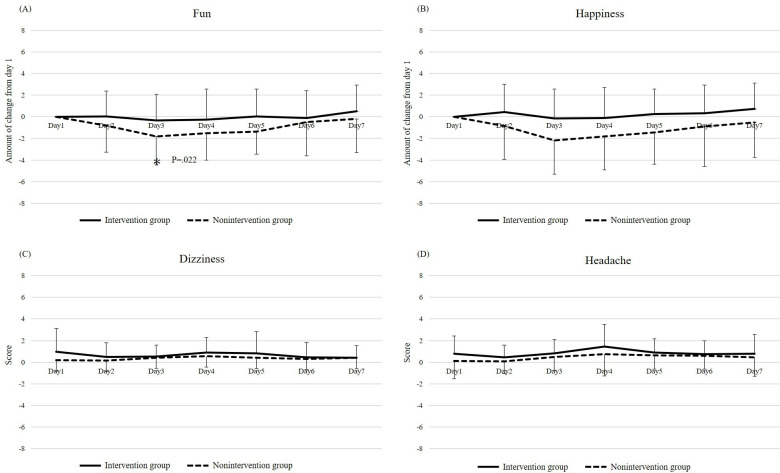
Amount of change in NRS of fun and happiness from day 1 (day X – day 1) (**A, B**), scores in NRS of dizziness and headache (**C, D**). *P<.05.

Figures 4A and 4B show the mean changes in NRS scores (days X–day 1) from days 2 to 7, relative to day 1. For fun, the change was significantly smaller (worse) on day 3 (*P*=.02) in the nonintervention group but not in the intervention group. For happiness, a significantly smaller change was observed on day 3 (*P*=.02) in the nonintervention group, but not in the intervention group. For dizziness and headache, the mean ESAS-r-J score did not exceed 2 for either group on any other day (Figure 4C and D).

### Change in Anxiety Before and After the First iVR Experience or Before and After Carboplatin Administration and Between Days 1 and 7

**Figure 5.**
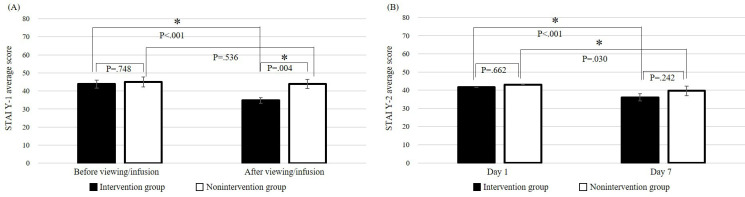
Changes in State-Trait Anxiety Inventory new questionnaire 1 and questionnaire 2 scores: (A) STAI Y-1. (B) STAI Y-2.

Figure 5A shows the mean STAI Y-1 scores before and after the first iVR experience on day 1, and before and after carboplatin administration in the nonintervention group. In the intervention group, the mean baseline score was 43.8, significantly decreasing to 34.8 post iVR (*P*<.001). In the nonintervention group, the scores were 44.9 at baseline and 43.9 postcarboplatin (*P*=.54). No significant difference was noted before VR viewing or infusion (*P*=.75); however, post iVR or infusion, the intervention group had a significantly lower mean score (*P*=.004, Figure 5A). The STAI Y-1 classifies anxiety into 5 levels, with state anxiety at ≥42 points and trait anxiety at ≥45 points considered high. Anxiety transitions on the STAI Y-1 showed 17 intervention participants had anxiety levels ≤2, and 11 had levels ≥3 before iVR, while 24 had levels ≤2, and 4 had levels ≥3 post iVR (*P*=.04). Figure 5B shows the mean STAI Y-2 scores on days 1 and 7. In the intervention group, the mean score dropped significantly from 41.6 on day 1 to 36.1 on day 7 (*P*<.001). The nonintervention group saw a decrease from 43.1 at baseline to 39.7 on day 7 (*P*=.03). Comparing STAI Y-2 scores between groups, no significant differences were found between days 1 and 7 (day 1: *P*=.66, day 7: *P*=.24).

### Perceptions After the iVR Experience on Day 7

To the question “How easy was it to learn how to operate this VR program?” 89.3% (25/28) of the respondents supported the simplicity of the iVR program, such as “It was very easy,” “I think I will be able to do it if I operate it several times,” and “It was easy enough to understand right away after listening to the explanation," among others. On the other hand, 10.7% (3/28) of the participants felt difficulty in operating the iVR program, such as “I am not good at operating the program, so I am glad if it is easy” and “It is a little difficult”. To the question “How confident would you be in recommending this VR program to a friend who is experiencing a similar situation?” 92.9% (26/28) of the respondents gave positive answers such as “I would definitely recommend it,” “I recommend it because I can forget about negative things while watching it,” and “I would definitely like them to experience it if they have a chance.” This was the most common response. Of these, 15.4% (4/26) of the participants gave answers that took their physical condition into consideration, such as “I would definitely recommend it if I did not feel bad,” and “It depends on my physical condition". The percentage of participants who did not respond to the questionnaire was 7.1% (2/28 participants). To the question “How useful do you think this program has been for you?”, 96.4% (27/28) of the participants responded positively to the usefulness of the iVR program, such as “It was good for a change of pace,” “I felt more comfortable,” and “It gave me more fun and led to new interests". To the question “Were there any difficulties you experienced during this session?”, 57.1% (16/28) of the participants answered “nothing in particular,” while 39.3% (11/28) reported that “the moving viewpoint made me a little drunk,” “the lens was foggy,” and “I felt too sick to experience it in the first place".

### IPOS-J Scores Before Administration and the Outpatient Conversion Rate for the Second Course

The mean values of the IPOS-J scores before the administration of the second course are shown in Table S3 in Multimedia Appendix 4. Only mean shortness of breath was significantly lower in the usual care group than in the intervention group (*P*=.02). The outpatient conversion rate for the second course was 35.7% (10/28 patients) in the intervention group and 3.3% (1/30 patients) in the nonintervention group; the intervention group had a significantly higher conversion rate (*P*=.002).

## Discussion

### Principal Findings

This study suggests that iVR may be more effective than nonintervention in reducing the deterioration of pain, depression, and nausea, especially by delaying the onset of nausea and speeding up recovery. The side effects and countermeasures of TC and TC+Bev therapy can be divided into acute and delayed phases. In the acute phase, infusion reactions, particularly to paclitaxel, are typical symptoms. Prophylactic administration of antihistamines and corticosteroids was used to treat infusion reactions. There was no difference in the incidence of acute side effects between the TC and TC+Bev therapy groups in patients with ovarian cancer [15]. In the delayed phase, side effects include nausea and vomiting, myelosuppression, myalgia/arthralgia, peripheral neuropathy, tiredness, constipation, and hair loss. In addition to these side effects, hypertension and proteinuria should be noted with TC+Bev therapy. Supportive care is effective for nausea and vomiting in both acute and delayed phases.

First, changes in the ESAS-r-J scores for nausea showed that in the intervention group, nausea worsened from baseline only on day 4, whereas it worsened on days 3, 4, and 5 in the nonintervention group. Furthermore, the number of patients who used additional antiemetic drugs during the delayed treatment phase was higher in the nonintervention group than that in the intervention group. Although the effect of iVR on nausea in patients with gynecological cancer has not been investigated, Wong et al [16] reported that iVR reduced nausea in pediatric cancer patients undergoing first-line chemotherapy, which is consistent with the findings of this study. Although no significant difference was observed in the late CR rate between the groups (64.3% in the intervention group and 40.0% in the nonintervention group), the difference of 24.3% meets the international standard of “10% or more improvement,” which is recognized as an indication of a new and effective treatment. Fujiwara et al [17] reported that among patients with gynecological cancer receiving TC therapy, the first- and second-cycle delayed CR rates were 65.8% in the triple therapy group treated with granisetron (a first-generation 5-HT3 receptor antagonist), aprepitant (a selective NK1 receptor antagonist), and dexamethasone. Hashimoto et al [18] evaluated the efficacy of olanzapine (5 mg) added to the triple therapy of palonosetron, aprepitant, and dexamethasone for delayed nausea and vomiting in patients receiving initial cisplatin therapy and reported that the delayed CR rate in the group receiving olanzapine was 79%, whereas that in the group receiving triple therapy was 66%. Compared with the CR rates in previous studies [17,18], the CR rate was similar in the intervention group and ≥20% lower in the nonintervention group in this study. However, in these 2 studies, dexamethasone was administered after day 2 (until day 3 in Fujiwara et al [17] study and until day 4 in Hashimoto et al [18] study), which was not the case in the present study. Therefore, we believe that the difference in the duration of dexamethasone administration likely contributed to the disparity in the CR rates. Hashimoto et al [18] reported that the addition of olanzapine to conventional supportive care for cisplatin administration improved the CR rate by 13% compared with conventional supportive care. In our study, iVR intervention also improved the CR rate by 24.3% compared with the nonintervention group, which may have clinical significance as a method to achieve a higher CR rate than conventional supportive care. In addition to supportive care, relaxation methods, such as deep breathing, meditation, and music therapy, have been recommended by the National Comprehensive Cancer Network (NCCN) guidelines for patients’ nausea and vomiting, 2022 [19]. We believe that the nonpharmacological approach of the iVR intervention could have provided a similar effect to that of relaxation, which is a nonpharmacological therapy.

Second, changes in ESAS-r-J scores for drowsiness showed that symptoms improved from day 1 to day 2 in the intervention group compared to day 2 to day 7 in the nonintervention group. TC or TC+Bev therapy is often problematic because the alcohol (anhydrous ethanol) in paclitaxel tends to cause drowsiness on the day of anticancer drug administration. In the nonintervention group, the day 1 scores were higher because of paclitaxel-induced drowsiness on the first day of TC or TC+Bev therapy, whereas in the intervention group, drowsiness was reduced by the iVR experience, which may have resulted in lower scores on the day of anticancer drug administration. Changes in the ESAS-r-J scores for pain showed that patients in the nonintervention group experienced significantly more pain on days 3 and 4 than on day 1, whereas those in the intervention group experienced more pain on day 4 than on day 1. For delayed pain, analgesics, such as NSAIDs, are mainly administered. In this study, many patients also abruptly took loxoprofen or acetaminophen tablets; however, there was no significant difference in the percentage of patients who took analgesics between the 2 groups. Since iVR has been reported to relieve pain in patients with cancer [20], the intervention group may have had fewer days of greater pain severity than the baseline group, compared to the nonintervention group.

Changes in ESAS-r-J scores for depression and tiredness revealed that patients in the nonintervention group were significantly more depressed and tired on day 4, whereas those in the intervention group did not experience significant worsening on any day compared to day 1. We have previously found that iVR temporarily improved pain, depression, and tiredness in patients with terminal cancer under palliative care [5], which is consistent with the results of this study. Regarding the mean change in NRS scores related to the positive feelings that can result from the iVR experience, patients in the intervention group did not have such experiences on any day, with significantly lower scores than on day 1, whereas patients in the nonintervention group had significantly lower scores on days 3 and 4 than on day 1. In addition, the intervention group had higher mean NRS scores of positive feelings than the nonintervention group on all days (days 1‐7), suggesting that the iVR experience was effective in preventing the decline of positive feelings. Schneider et al [21] reported that iVR may reduce negative emotions and induce positive emotions; a similar effect was demonstrated in this study. In addition, as relaxation has been reported to be effective in reducing depression and fatigue in patients with cancer [22,23], we believe that the intervention group may have been able to reduce these increases.

Third, regarding the iVR experiences-induced adverse effects, no significant difference was observed between the 2 groups for dizziness or headache, suggesting that the adverse reactions associated with iVR experiences may not be problematic. Although VR sickness is often a concern in VR experiences [24], in this study, the duration of the iVR experience was set to 10 minutes, and avoiding images in which the viewpoint moved independently of the patient will likely prevent adverse reactions.

Fourth, the changes in STAI Y-1 scores before and after the iVR experience suggest that the iVR experience was effective in temporarily reducing anxiety. The mean STAI Y-1 score in the intervention group significantly decreased after the iVR experience. However, no significant difference was observed in the nonintervention group, and the mean score after the iVR experience (after administration) was significantly lower in the intervention group. Furthermore, when anxiety levels were classified based on the STAI Y-1 scores, the number of patients with high anxiety levels decreased significantly after the iVR experience in the intervention group, whereas no significant difference was observed in the nonintervention group. These results are consistent with those of Fabi et al [25] who reported a significant reduction in anxiety after a VR experience in patients with early-stage breast and ovarian cancers undergoing chemotherapy. In contrast, the mean STAI Y-2 score on day 7 was significantly lower than that on day 1 in both the intervention and nonintervention groups; however, no significant difference was observed between the two groups on days 1 and 7. In addition, when anxiety levels were classified based on STAI Y-2 scores, no significant differences were observed between the 2 groups. These results suggest that iVR intervention is more effective in reducing state anxiety than trait anxiety. The significant decrease in anxiety in both groups and the lack of a significant difference between the 2 groups are unclear, but they are believed to be attributable to factors other than iVR, such as the approaching discharge date and the decrease in side effects of TC or TC+Bev therapy.

Fifth, no significant difference in ESAS-r-J or IPOS-J scores was observed for the second course, indicating that the iVR experience had no effect on anticipatory physical and mental symptoms or other symptoms at initial admission. However, the intervention group showed a significantly higher rate of transition to outpatient care during the second course. The criteria for transfer to an outpatient setting usually include the absence of serious side effects during initial chemotherapy, side effects requiring hospitalization during subsequent courses of treatment [26]. In addition, the patient’s desire for outpatient transition. Although we did not investigate outpatient transition in this study, the results suggest that the iVR experience may be a factor in facilitating smooth outpatient transition.

### Limitations

This study had some limitations. First, the different number of visits made by the researchers to the patients in the intervention and nonintervention groups introduced interpersonal bias. Patients in the intervention group responded to the questionnaire in an interpersonal situation because they visited daily from days 1 to 7 to experience iVR. Conversely, patients in the nonintervention group visited only twice, once when the questionnaire was distributed on day 1 and the other when it was collected on day 7. The difference in the number of visits and response statuses may have affected the way the patients scored the questionnaires. Second, because all evaluations in this study were based on a questionnaire, they relied on patients’ subjectivity and did not provide any insight into the objective evaluation or effects of iVR. Third, the participants in the comparison group received nonintervention, which precluded a direct comparison with interventions using 2D images, such as TV. However, it would be interesting to explore the potential differences in the effectiveness of 2D and iVR images. Although we did not directly compare these modalities, we believe that the effect may be attributable to their ability to create the sense of immersion that VR produces. Schutte et al [27] compared the effects of iVR to 2D monitors on the empathy and immersion of “being there” and reported that both empathy and immersion are stronger with iVR than with 2D monitors. Furthermore, Austin et al [28] conducted a crossover study comparing the pain reduction effects of 2D and iVR in inpatients receiving palliative care and found that iVR was significantly more immersive and that increased immersion was associated with pain reduction. Fourth, in this study, patients were asked to experience content according to their own preferences to avoid placing the burden of experiencing content that they were not interested in. Therefore, the iVR content experienced by the patients varied from patient to patient, and we were unable to examine the differences based on the content. However, based on patient feedback, they enjoyed the iVR experience when the content they experienced matched their preferences (eg, those who liked Disney were excited about the VR app that allowed them to enter Disney movies). Therefore, while working to identify content that will be acceptable to many people, providing content that practically matches patient preferences may be considered an important factor in the future. Fifth, we set the duration of each iVR viewing to approximately 10 minutes to avoid the risk of VR sickness and the intervention period to 7 days based on our experience that patients undergoing initial TC or TC+Bev therapy are hospitalized for approximately 7 days; however, it is unclear whether this is the optimal duration of the iVR experience and intervention period. In fact, we received feedback from patients that 10 minutes of viewing was too short; therefore, we believe that adjusting the duration of iVR viewing according to their wishes to the extent that they did not experience VR sickness may increase the effectiveness of the intervention. In addition, several patients commented on the seventh day of the intervention, “Is the period when I can experience iVR already over? I am going to miss it from tomorrow.” Therefore, the effectiveness of the iVR intervention could be enhanced by adjusting the intervention period according to the patients’ wishes. Although our intervention was limited to inpatients, there is a report that video games can improve well-being even when viewed at home [29]; therefore, we believe that iVR experiences at home may have similar effects to those during hospitalization. Sixth, the use of many assessment measures may have reduced the statistical power. Since this is the first study in the world to observe the impact of iVR intervention on the side effects of TC or TC+Bev therapy for seven consecutive days, we used multiple assessment measures to be able to pick up changes in various side effects. Since this study has revealed trends in the impact of iVR intervention on each side effect, it is necessary to refer to this study in the future to narrow down the target side effects and assessment measures to evaluate them, and design a study with an accurate sample size.

### Strengths

Given that few studies worldwide have examined the effects of iVR interventions over a 7-day period, this study provides new insights.

### Conclusion

The iVR experience may reduce the deterioration of nausea and depression in patients with gynecologic cancer undergoing TC or TC+Bev therapy and contribute to positive feelings of fun and happiness. As the management of anxiety has been reported to help prevent chemotherapy-induced nausea and vomiting [30], although further studies are needed, we believe that we have proposed a new treatment method that uses iVR to reduce anxiety and prevent delayed nausea while providing patients with positive emotions.

## Supplementary material

10.2196/65924 Multimedia Appendix 1 A prepared pamphlet for the immersive virtual reality experience.

10.2196/65924 Multimedia Appendix 2 Virtual reality apps used in this study.

10.2196/65924 Multimedia Appendix 3 Additional antiemetic drugs used in each group.

10.2196/65924 Multimedia Appendix 4 Comparison of patient characteristics and 7-day recall integrated palliative care outcome scale Japanese version.

10.2196/65924 Checklist 1 CONSORT (Consolidated Standards of Reporting Trials) 2025 checklist.

## Abbreviations

CONSORT: Consolidated Standards of Reporting Trials
CR: complete response
DTx: digital therapeutics
ECOG PS: Eastern Cooperative Oncology Group Performance Status
ESAS-r-J: Japanese version of the revised Edmonton Symptom Assessment System
IPOS-J: Japanese version of the integrated palliative outcome scale
iVR: immersive virtual reality
NCCN: National Comprehensive Cancer Network
NRS: numerical rating scale
NSAID: non-steroidal anti-inflammatory drug
STAI: State-Trait Anxiety Inventory
TC: paclitaxel-carboplatin
TC+Bev: paclitaxel-carboplatin plus bevacizumab
VR: virtual reality
WHO: World Health Organization
