# Supplementary figures and images for: Effect of Immersive Virtual Reality on Chemotherapy-Related Side Effects in Patients Receiving Paclitaxel-Carboplatin With or Without Bevacizumab: 2-Arm Randomized Controlled Trial
Source: J Med Internet Res. 2025 Aug 14;27:e65924. doi: 10.2196/65924 (PMC12352699; doi:10.2196/65924)

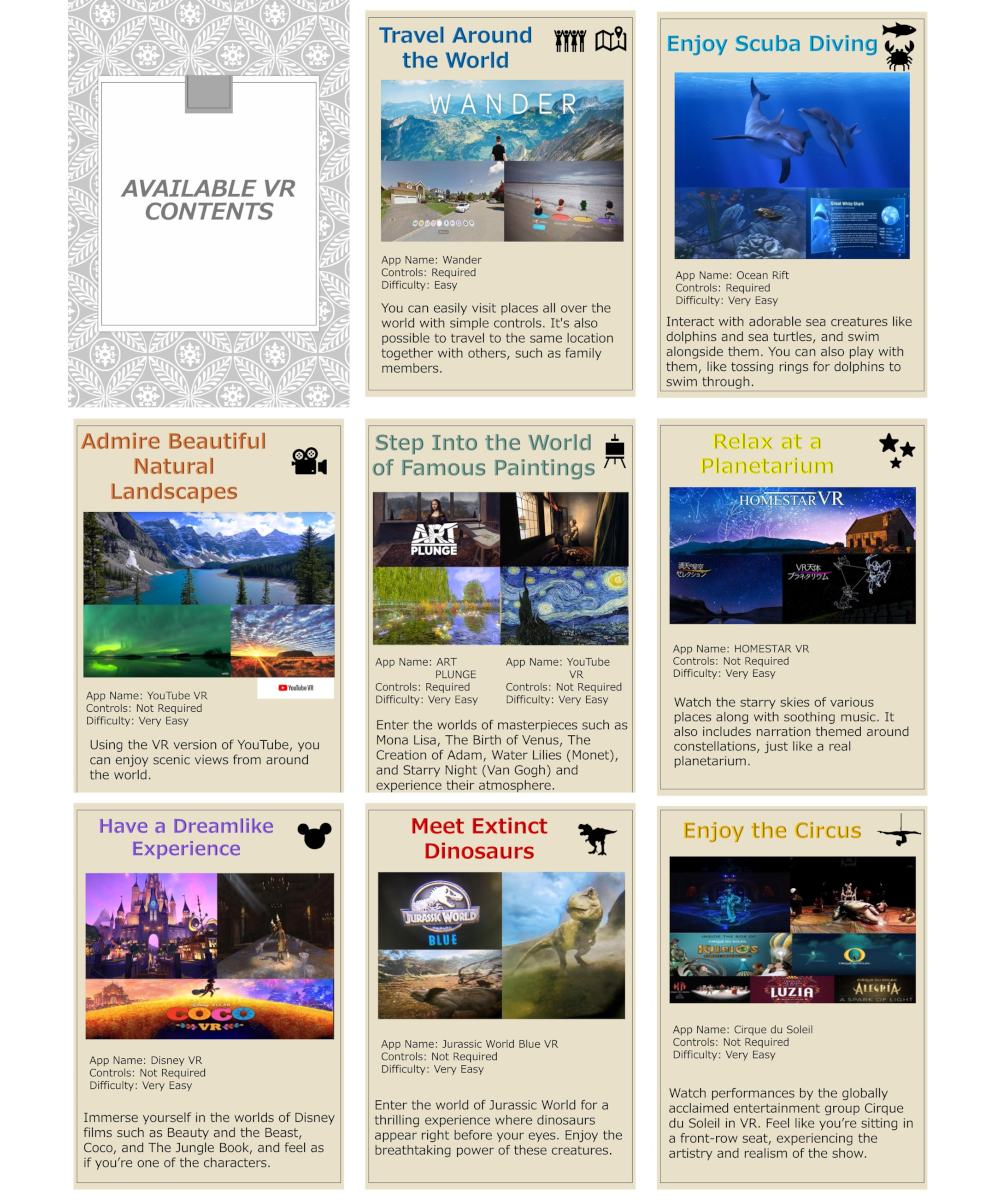

Supplement: Multimedia Appendix 1 [file jmir-v27-e65924-s001.png]
