# Supplementary material for: Effect of Immersive Virtual Reality on Chemotherapy-Related Side Effects in Patients Receiving Paclitaxel-Carboplatin With or Without Bevacizumab: 2-Arm Randomized Controlled Trial
Source: J Med Internet Res. 2025 Aug 14;27:e65924. doi: 10.2196/65924 (PMC12352699; doi:10.2196/65924)
Supplement: Multimedia Appendix 2 [file jmir-v27-e65924-s002.docx]

Supplemental table 1. VR apps used in this study

| **VR apps name** | **Content offered** |
| --- | --- |
| Wander | VR travel to various destinations worldwide |
| Ocean Rift | Interaction with sea creatures (dolphins, manatees, etc.) |
| YouTube VR | 360-degree video of natural scenery |
| HOMESTAR VR | Experience starry skies and planetariums worldwide |
| ART PLUNGE | 3D experience of famous paintings (Mona Lisa, Starry Night, etc.) |
| Disney VR | Experience scenes from Disney movies (Beauty and the Beast, etc.) |
| Jurassic World: Blue | VR experiences based on the movie "Jurassic World" |
| Cirque du Soleil VR | Experience 5 representative Cirque du Soleil performances |
