# Supplementary material for: Effect of Immersive Virtual Reality on Chemotherapy-Related Side Effects in Patients Receiving Paclitaxel-Carboplatin With or Without Bevacizumab: 2-Arm Randomized Controlled Trial
Source: J Med Internet Res. 2025 Aug 14;27:e65924. doi: 10.2196/65924 (PMC12352699; doi:10.2196/65924)
Supplement: Multimedia Appendix 3 [file jmir-v27-e65924-s003.docx]

| **Intervention group** | **Conventional treatment group** |
| --- | --- |
| Metoclopramide tablet (5 mg) | Olanzapine OD tablet (2·5 mg) |
|  | Olanzapine OD tablet (5 mg) |
|  | Nauzelin OD tablet (10 mg) |
|  | Metoclopramide tablet (5 mg) |

Supplemental table 2. Additional antiemetic drugs used in each group
