# Supplementary material for: Effect of Immersive Virtual Reality on Chemotherapy-Related Side Effects in Patients Receiving Paclitaxel-Carboplatin With or Without Bevacizumab: 2-Arm Randomized Controlled Trial
Source: J Med Internet Res. 2025 Aug 14;27:e65924. doi: 10.2196/65924 (PMC12352699; doi:10.2196/65924)
Supplement: Multimedia Appendix 4 [file jmir-v27-e65924-s004.docx]

Supplemental table 3. Comparison of patient characteristics and 7 day recall integrated palliative care outcome scale Japanese version

|  | **Intervention group (n=25)** | **Usual treatment group (n=23)** | **p-value** |
| --- | --- | --- | --- |
| Pain | 1·0 (0·0–1·0) | 1·0 (0·0–1·0) | 0·8174 |
| Shortness of breath | 1·0 (0·0–1·0) | 0·0 (0·0–0·0) | 0·0235 |
| Weakness or lack of energy | 1·0 (1·0–2·0) | 1·0 (0·5–2·0) | 0·6177 |
| Nausea (feeling like you are going to be sick) | 0·0 (0·0–0·0) | 0·0 (0·0–0·5) | 0·5831 |
| Vomiting (being sick) | 0·0 (0·0–0·0) | 0·0 (0·0–0·0) | 0·658 |
| Poor appetite | 0·0 (0·0–1·0) | 0·0 (0·0–0·0) | 0·5089 |
| Constipation | 0·0 (0·0–1·0) | 0·0 (0·0–1·0) | 0·6496 |
| Sore or dry mouth | 0·0 (0·0–1·0) | 0·0 (0·0–1·0) | 0·6133 |
| Drowsiness | 0·0 (0·0–1·0) | 0·0 (0·0–1·0) | 0·6899 |
| Poor mobility | 1·0 (0·0–2·0) | 1·0 (0·0–2·0) | 0·8631 |
| Have you been feeling anxious or worried about your illness or treatment? | 1·0 (1·0–3·0) | 2·0 (1·0–2·0) | 0·6463 |
| Have any of your family or friends been anxious or worried about you? | 2·0 (1·0–3·0) | 2·0 (1·0–2·0) | 0·2209 |
| Have you been feeling depressed? | 1·0 (1·0–2·0) | 2·0 (0·5–2·5) | 0·3804 |
| Have you felt at peace? | 1·0 (0·0–1·0) | 1·0 (1·0–2·0) | 0·339 |
| Have you been able to share how you are feeling with your family or friends as much as you wanted? | 1·0 (0·0–2·0) | 2·0 (0·0–3·0) | 0·4996 |
| Have you had as much information as you wanted? | 1·0 (0·0–2·0) | 0·0 (0·0–3·0) | 0·7119 |
| Have any practical problems resulting from your illness been addressed? (such as financial or personal) | 1·0 (0·0–1·0) | 1·0 (0·0–1·0) | 0·9116 |
